# Emotion Recognition Ability Test Using JACFEE Photos: A Validity/Reliability Study of a War Veterans' Sample and Their Offspring

**DOI:** 10.1371/journal.pone.0132293

**Published:** 2015-07-06

**Authors:** Ivone Castro-Vale, Milton Severo, Davide Carvalho, Rui Mota-Cardoso

**Affiliations:** 1 Medical Psychology Unit, Department of Clinical Neurosciences and Mental Health, Faculty of Medicine, University of Porto, Porto, Portugal; 2 Department of Clinical Epidemiology, Predictive Medicine and Public Health, Faculty of Medicine, University of Porto, Porto, Portugal; 3 Department of Medical Education and Simulation, Faculty of Medicine, University of Porto, Porto, Portugal; 4 Department of Endocrinology, Diabetes and Metabolism, Centro Hospitalar Sāo Joāo, Faculty of Medicine, University of Porto, Porto, Portugal; 5 Instituto de Investigação e Inovação em Saúde, Universidade do Porto, Porto, Portugal; Liaoning Normal University, CHINA

## Abstract

Emotion recognition is very important for social interaction. Several mental disorders influence facial emotion recognition. War veterans and their offspring are subject to an increased risk of developing psychopathology. Emotion recognition is an important aspect that needs to be addressed in this population. To our knowledge, no test exists that is validated for use with war veterans and their offspring. The current study aimed to validate the JACFEE photo set to study facial emotion recognition in war veterans and their offspring. The JACFEE photo set was presented to 135 participants, comprised of 62 male war veterans and 73 war veterans’ offspring. The participants identified the facial emotion presented from amongst the possible seven emotions that were tested for: anger, contempt, disgust, fear, happiness, sadness, and surprise. A loglinear model was used to evaluate whether the agreement between the intended and the chosen emotions was higher than the expected. Overall agreement between chosen and intended emotions was 76.3% (Cohen kappa = 0.72). The agreement ranged from 63% (sadness expressions) to 91% (happiness expressions). The reliability by emotion ranged from 0.617 to 0.843 and the overall JACFEE photo set Cronbach alpha was 0.911. The offspring showed higher agreement when compared with the veterans (RR: 41.52 vs 12.12, p < 0.001), which confirms the construct validity of the test. The JACFEE set of photos showed good validity and reliability indices, which makes it an adequate instrument for researching emotion recognition ability in the study sample of war veterans and their respective offspring.

## Introduction

Emotions are inherent to human nature. They exist because we need to recognize and respond to significant events that are related to survival and/or the maintenance of well-being, and thus they serve several functions [1]. Emotions are common to other animals, particularly vertebrates [2]. They are brought about by stimulus presentation, being comprised of such processes as arousal, appraisal, production of an affective state, as well as behavior and respective regulation [3]. Basic emotions can be distinguished from each another and are the result of evolution developing the best way to deal with fundamental life tasks [4]. The theme of an emotion is influenced phylogenetically and variations to that theme are influenced in turn by social experience [4].

Basic emotions are considered to be universal, because they have been shown to be categorized by different cultures and ethnicities [5, 6]. Emotions have distinctive behavior patterns and some are known to involve specific neuronal structures, as evidenced by stimulation, lesion and functional neuroimaging studies. For example, fear is known to depend in great part on the amygdala [7, 8] and disgust involves the insula [9].

Basic emotions have been shown to have specific autonomic nervous system responses [10]. For example, in the case of fear, blood flow is directed to the legs to prepare for flying away from the dangerous stimulus. Interestingly, when a person is asked to create a facial expression of fear, the same autonomic response emerges, evidencing the strong emotional pattern that has been phylogenetically developed [11, 12].

Facial expressions are an important component of emotional behavior and are specific to basic emotions [13]. Facial emotion recognition is essential for social interaction, as well as for clinical communication. Facial expressions have been extensively used to study emotions’ neurobiology, such as the amygdala’s role in social judgments [14], and many other structures as well [15]. Neuroimaging studies also suggest that different neural circuits underlie each facial emotion perception [8].

Facial emotion recognition is known to diminish with age [16–20], and women have been reported to have better accuracy in identifying facial expressions of emotion than men [19, 21, 22]. Literacy has also been found to positively influence facial emotion recognition [19].

Many clinical situations limit facial emotion recognition, such as psychiatric disorders and some neurologic disorders. Depression and bipolar disorder are associated with moderate and stable deficiency in facial emotion perception, moderated by self-reported depression, age at time of testing, and years of education [23]. Facial emotion recognition ability is impaired in patients with schizophrenia, [24, 25] and is probably a characteristic trait of this illness [26]. Anxiety disorders have also been associated with impairment in facial emotion recognition—patients with panic disorder showed an inability to recognize disgust and fear and register higher accuracies in recognizing surprise [27]. Patients with post-traumatic stress disorder (PTSD) showed reduced accuracy and sensitivity to recognize facial expressions of fear and sadness [28]. Personality disorders, such as schizotypal and psychopathic, have also been associated with impairments in recognizing facial expressions of emotion [29, 30]. Autism spectrum disorders have been extensively studied with regards to facial emotion recognition and global impairment in identifying emotions has been particularly found in the case of negative ones [31, 32]. Neurologic disorders, such as Huntington’s disease [33] and Parkinson’s disease [34], have shown specific emotion recognition deficiency with respect to disgust and sadness.

Several methodologies have been used to study facial emotion recognition. In the literature we can find many photo sets and videos for this purpose. Some sets include only one ethnicity, whilst others include hundreds of stimuli [35], and others even use computerized faces [36] or morphed faces [37, 38].

One of the most important and frequently used methodologies is the set of photos developed by Matsumoto and Ekman—the Japanese and Caucasian Facial Expressions of Emotion (JACFEE) [39]. It is comprised of 56 photos of different posers from two different ethnicities, with an equal number of Japanese and Caucasian represented, and an equal number of females and males, with eight photos for each of the seven basic emotions (anger, contempt, disgust, fear, happiness, sadness, and surprise). All of the facial emotions have been scored by the Facial Action Coding System [40], to ensure that the expressions were exactly the ones intended. JACFEE photos have been validated and have shown multi-culture reliability [5].

JACFEE photos have been used in several research fields, such as: Basic emotion research neurobiology in normal subjects [17, 41]; psychiatric disorders; schizophrenia [25, 42]; anxiety disorders [27] and personality disorders [29, 43].

War veterans are a unique population, as they have been exposed to traumatic events and are at risk of developing psychiatric disorders [44], such as PTSD and other anxiety disorders, mood disorders and substance dependent disorders, all of which are conditions that have been associated with deficits in emotion recognition ability. Their offspring are also at an increased risk of psychopathology [45, 46] and are also an important population for study purposes, as, for example, there are some reports of altered facial emotion recognition in normal offspring of depressed patients [47–49].

Portugal was involved in colonial wars from 1961 to 1974, during which 1 million Portuguese solders were involved. This has resulted in psychopathology, both in veterans [50] and also in their offspring [51].

To our knowledge, there are no reliability or validity studies of veterans’ populations and their offspring for tests of facial emotion recognition. A valid and reliable test for veterans and their offspring is needed, which can be used in future studies that attempt to control for hereditary problems that influence facial emotional recognition, or studies that want to show that problems influencing facial emotional recognition are transferred across generations. To carry out this type of studies we need a test which is valid and reliable in both groups. Our aim was to study the JACFEE photo set reliability/validity for a sample of veterans from the Portuguese colonial wars and also that of their offspring. We show that the JACFEE set of photos has good validity and reliability indices in the study sample of war veterans and their respective offspring.

## Material and Methods

### Ethics Statement

This study was approved by the Ethics Committee of the author’s university (Comissão de Ética para a Saúde do Centro Hospitalar São João / Faculdade de Medicina da Universidade do Porto). All participants received a written and verbal description of the study and gave their written informed consent.

### Participants

Data are presented as the mean (standard deviation). Sixty two white veterans [mean age = 65.31 (3.71) years] of the Portuguese colonial wars were enrolled for the study. The sampling procedure used two ways of selecting participants: 75.8% from an outpatient clinic of the Portuguese Disabled Veterans Association (ADFA), and 24.2% from three lists of veterans’ companies from war time. Half of the veterans (51.61%) were disabled, and all were outpatients. Women did not participate in the Portuguese colonial wars as combatants. Offspring (n = 73; 54.1%) from 46 veterans were also included [mean age = 35.66 (4.82) years; woman: 60.3%)]. The mean age of the total sample (n = 135) was 49.27 (15.41) years’ old, and the median education in years (inter-quartile range) was 9 (4–15). Veterans’ median education was 4 (4–9.5) years, and offspring’s median education was 12 (9–15) years. All participants were of European descent.

### Task

In a university setting, participants identified the emotion expressed in 56 photos from the JACFEE set [39], balanced for emotion and gender. Photos were displayed on a computer screen (after being controlled for brightness) in random order, with no successive photos belonging to the same emotion and the order of presentation was kept constant between subjects. During this task, participants chose a single term from a list of seven emotion names that best described the emotion expressed by the poser in the photo. The time taken to complete the task was measured in minutes. Because of the age of the veterans, a choice was made not to restrict the time of each photo presentation, neither to measure the intensity of emotions. Socio-demographic data was then collected.

### Statistical methods

We used relative and absolute frequency of chosen emotions per intended emotional expression. The Cronbach alpha was used to evaluate the internal consistency. See Table 1.

**Table 1:** Example of the data collapsed according to the frequencies of the chosen and intended expression categories for each individual.

ID	Chosen	Intend	Frequency
1	Anger	Anger	7
1	Contempt	Anger	0
1	Disgust	Anger	1
1	Fear	Anger	0
1	Happiness	Anger	0
1	Sadness	Anger	0
	…	…	…

A generalized linear Poisson model with log link was used to evaluate whether the agreement between the intended and chosen emotions was higher than the expected, assuming independence between chosen and intended emotions [52]. Furthermore, these models were used to assess whether the agreement was higher in offspring, higher educated, younger and female gender, than in veterans, lower educated, older and male gender. These comparisons were done to access the construct validity of the test. The magnitude of the agreement was measured using relative risk and the respective 95% confidence interval.

## Results

For each image, we calculated how many participants chose the target emotion (See Table 2). The overall agreement between chosen and intended emotions was 76.3%, and the Cohen kappa was 0.72. The agreement ranged from 63% (sadness expressions) to 91% (happiness expressions). When we stratify by offspring and veterans, the overall agreement was lower for veterans when compared to their offspring, for all the emotions studied, as shown in Table 3.

**Table 2:** Number and Percentage of Chosen Emotions per Intended Emotional Expression.

Pooled							
	Chosen Expression						
	Anger	Contempt	Disgust	Fear	Happiness	Sadness	Surprise
	N (%)	N (%)	N (%)	N (%)	N (%)	N (%)	N (%)
**Intended Expression**							
Anger	910 (84.8)	30 (2.8)	28 (2.6)	44 (4.1)	3 (0.3)	16 (1.5)	42 (3.9)
Contempt	29 (2.7)	791(73.6)	25 (2.3)	21 (2.0)	26 (2.4)	105 (9.8)	78 (7.3)
Disgust	90 (8.4)	109 (10.1)	771 (71.7)	22 (2.0)	2 (0.2)	49 (4.6)	32 (3.0)
Fear	60 (5.6)	14 (1.3)	59 (5.5)	687 (64.0)	9 (0.8)	27 (2.5)	218 (20.3)
Happiness	7 (0.7)	7 (0.7)	2 (0.2)	5 (0.5)	978 (91.3)	21 (2.0)	51 (4.8)
Sadness	19 (1.8)	159 (14.8)	31 (2.9)	58 (5.4)	8 (0.7)	676 (63.0)	122 (11.4)
Surprise	10 (0.9)	19 (1.8)	16 (1.5)	91 (8.5)	7 (0.7)	6 (0.6)	926 (86.1)

**Table 3:** Number and Percentage of Chosen Emotions per Intended Emotional Expression, Stratified by Veterans and Offspring.

Veterans							
	Chosen Expression						
	Anger	Contempt	Disgust	Fear	Happiness	Sadness	Surprise
	N (%)	N (%)	N (%)	N (%)	N (%)	N (%)	N (%)
**Intended Expression**							
Anger	364 (74.4)	16 (2.8)	25 (5.1)	30 (6.1)	3 (0.6)	12 (2.5)	39 (0.8)
Contempt	24 (4.9)	292 (59.5)	12 (2.4)	20 (4.1)	15 (3.1)	67 (13.6)	61 (12.4)
Disgust	36 (7.3)	70 (14.3)	296 (60.3)	19 (3.9)	2 (0.4)	41 (8.4)	27 (5.5)
Fear	58 (11.8)	11 (2.2)	25 (5.1)	265 (54.0)	2 (0.4)	16 (3.3)	114 (23.2)
Happiness	5 (1.0)	6 (1.2)	2 (0.4)	2 (0.4)	426 (87.3)	15 (3.1)	32 (6.6)
Sadness	17 (3.5)	105 (21.4)	22 (4.5)	41 (8.4)	5 (1.0)	197 (40.2)	103 (21.0)
Surprise	6 (1.2)	13 (2.6)	13 (2.6)	61 (12.4)	6 (1.2)	6 (1.2)	386 (78.6)
**Offspring**							
**Intended Expression**							
Anger	546 (93.5)	14 (2.4)	3 (0.5)	14 (2.4)	0 (0.0)	4 (0.7)	3 (0.5)
Contempt	5 (0.9)	499 (85.4)	13 (2.2)	1 (0.2)	11 (1.9)	38 (6.5)	17 (2.9)
Disgust	54 (9.2)	39 (6.7)	475 (81.3)	3 (0.5)	0 (0.0)	8 (1.4)	5 (0.9)
Fear	2 (0.3)	3 (0.5)	34 (5.8)	422 (72.4)	7 (1.2)	11 (1.9)	104 (17.8)
Happiness	2 (0.3)	1 (0.2)	0 (0.0)	3 (0.5)	552 (94.7)	6 (1.0)	19 (3.3)
Sadness	2 (0.3)	54 (9.3)	9 (1.5)	17 (2.9)	3 (0.5)	479 (82.2)	19 (3.3)
Surprise	4 (0.7)	6 (1.0)	3 (0.5)	30 (5.1)	1 (0.2)	0 (0.0)	540 (92.5)

A close look at Table 2 reveals that, for some expressions, responses were not equally distributed across the chosen expressions: Faces with intended fear were sometimes confused with surprise (20.3%), and vice versa (8.5%). Intended sadness was sometimes confused with either contempt (14.8%), or surprise (11.4%), and intended contempt was sometimes mistaken for either sadness (9.8%), or surprise (7.3%) (See Table 2). For the pooled sample, the reliability by emotion ranged from 0.617 to 0.843 (See Table 4), and the overall reliability for the JACFEE photo set between images was 0.911. Overall reliability stratified by veterans and offspring was 0.905 and 0.837, respectively.

**Table 4:** Reliability between Images of Each Intended Expression, Pooled and Stratified by Veterans and Offspring.

	Cronbach alpha		
Intended Expression	Pooled	Veterans	Offspring
Anger	0.806	0.799	0.627
Contempt	0.843	0.790	0.832
Disgust	0.714	0.672	0.654
Fear	0.657	0.688	0.491
Happiness	0.617	0.688	0.406
Sadness	0.777	0.612	0.545
Surprise	0.759	0.724	0.746
Overall	0.911	0.905	0.837

The frequencies of the categories in cases where the chosen expression equals the intended expression (agreement categories) was 21.4 (95% CI: 20.2 to 22.7) times higher in comparison to the categories where intended expression was different from chosen expression (disagreement categories), assuming the independence between both factors (Table 5). Table 5 shows an interaction between offspring and veterans in the agreement categories (p < 0.001). The offspring showed 41.53 (95% CI: 37.71 to 45.74) times higher frequencies in the agreement categories when compared with the disagreement categories whilst the veterans had only 12.12 (95% CI: 11.26 to 13.06) times higher frequencies. After stratifying by veterans and offspring we found that higher education (p < 0.001) and younger age (p < 0.001) were associated with higher agreement in both groups, and in the offspring group, female gender was associated with higher agreement (p < 0.001) than male gender (Table 6).

**Table 5:** Loglinear Poisson Model for the Frequencies of Chosen and Intended Expression Categories for Each Individual, Adjusted for Expected Frequencies, Assuming Independence between Both Factors.

	RR (95% CI)	p*
**Total**	21.4 (20.2; 22.7)	
Offspring	41.53 (37.71; 45.74)	< 0.001
Veterans	12.12 (11.26; 13.06)	

RR = relative risk; CI = confidence interval;

* p value for the interaction.

**Table 6:** Loglinear Poisson Model for the Frequencies of Observed and Intended Expression Categories for Each Individual, Adjusted for Expected Frequencies, Assuming Independence between Both Factors. Stratified by Education, Age, and Gender.

	Offspring	p *		Veterans	p *
	RR (95% CI)			RR (95% CI)	
Education (years)					
≤ 9	13.74 (12.45; 15.16)	< 0.001		13.36 (12.02; 14.84)	< 0.001
> 9	37.31 (32.69; 42.58)			39.17 (33.97; 45.17)	
Age (years)					
≤ 36	48.47 (40.12; 58.55)	< 0.001	≤ 65	24.41 (22.16; 26.90)	< 0.001
> 36	16.50 (15.14; 17.99)		> 65	12.45 (10.53; 14.72)	
Gender					
Female	46.79 (39.17; 55.79)	< 0.001		#	
Male	16.02 (14.66; 17.50)			#	

RR = relative risk; CI = confidence interval;

* p value for the interaction;

^#^ not shown because all veterans were male.

## Discussion

In this study, the 56 photos from the JACFEE set [39] were validated for a sample of veterans of the Portuguese colonial wars and their offspring. The photos were evaluated for their emotional content. The overall agreement between chosen and intended emotions was 76.3%, which is comparable to the multicultural agreement percentages found with this set of photos, which vary between 76.1% and 86.6% [5]. The kappa value for the agreement rates can be considered to be substantial [53]. The overall agreement was superior to other sets of photos [35, 54]. Happiness had the highest agreement level, followed by surprise, which replicated the findings of Biehl et al [5], Dodich et al [19] and Langner et al [55]. Happiness is consistently found to be the emotion that has the highest agreement level across studies [5, 19, 35, 54, 55]. The lowest agreement level was found for sadness, followed by fear. This finding was also reported by Elfenbein et al [54]. Goeleven et al [35], when using the unbiased hit rate, found that sadness was also the lowest emotion correctly recognized, except for fear.

There were systematic patterns of disagreement for fear, surprise, content and sadness. These patterns are similar to those found by Goeleven et al [35] and Langner et al [55]. Lower agreement for facial expressions of content may be related to problems of the expression label [56]. As for fear, it has been argued that this has characteristics of surprise [5].

Since facial emotion recognition can be considered as a multiple effect indicator construct model, reliability analysis through Cronbach alpha is adequate [57]. For the overall expressions, the Cronbach alpha, according to the Nunnally criterion [58], is excellent, although for intended expressions of happiness and fear, it is questionable. For the other emotions it is acceptable (sadness, surprise and disgust), and good (anger and contempt). When we stratified by veterans and offspring, the latter showed lower alpha values for the intended expressions of fear, happiness and sadness, due to the low variability between the offspring, which is shown by the high agreement frequencies.

We found that offspring had significantly higher frequencies in agreement categories when compared to the veterans. To our knowledge, no studies exist which compare the performance of parents with their adult offspring in facial emotion recognition. However offspring are expected to have better performances. This is probably accounted for by the lower age, higher education, and the inclusion of women in the offspring group, which are factors that have all been reported to positively influence facial emotion recognition ability [16–19, 21]. In fact, the literature shows that young adults, together with those with higher education have better performances than the elderly and those with lower education respectively [19, 20]. Moreover, our offspring sample included women, who have also been found to have better performances in emotion recognition [19, 21, 22]. Our results confirm these previous assumptions, which thus confirm the construct validity of the test.

Limitations: the participants were forced to choose between limited emotional term options, which might have biased the choice that they really would have made. However, this methodology is in accordance with the one used to validate this photo set and it relies on the rigorous construction of the set, and on the universality of these basic emotions [5]. Other factors which may influence emotion recognition, such as symmetry, have been studied previously using the JACFEE set of photos, and these concluded that they are mostly symmetrical [59].

Some researchers still use the earlier Ekman and Friesen’s Pictures of Facial Affect [19, 26], but this set has been reported to be superseded, which limits its ecological validity [35, 60]. Some picture databases are too long, with difficult tasks to perform. The JACFEE photo set is well balanced, in that it has a sufficient number of stimuli, and that its tasks are not too difficult to perform. This makes it a good instrument for the study of facial emotion recognition among elderly individuals.

In conclusion, for the first time, this research replicated in a sample of war veterans and their respective offspring the results of previous studies which aimed to validate instruments for evaluation of facial emotion recognition in non-clinical samples. The JACFEE set of photos showed good validity and reliability indices, and seems to be a valid instrument for investigating the recognition of facial emotions in populations of war veterans and their offspring. We think that now this test can be used in future studies that aim to control for hereditary problems that influence facial emotional recognition, or those that want to show that problems influencing facial emotional recognition are transferred across generations. We are now going to use this same test to characterize the influence of war-related PTSD on facial emotion recognition ability, and also the role of inheritance with regards to the interaction between these variables. Emerging questions of trauma inheritance and how it relates to psychopathology and social interaction need to be further investigated. The JACFEE photo set seems to be an adequate instrument for this purpose.
